# Primate-specific spliced *PMCHL *RNAs are non-protein coding in human and macaque tissues

**DOI:** 10.1186/1471-2148-8-330

**Published:** 2008-12-09

**Authors:** Sandra Schmieder, Fleur Darré-Toulemonde, Marie-Jeanne Arguel, Audrey Delerue-Audegond, Richard Christen, Jean-Louis Nahon

**Affiliations:** 1 Université de Nice-Sophia Antipolis, CNRS, Institut de Pharmacologie Moléculaire et Cellulaire, 660 route des lucioles, 06560 Valbonne, France; 2 Biothèque Primates/Primatech, Centre National de la Recherche Scientifique, Life Science Department, Bordeaux-Valbonne, France; 3 Université de Nice-Sophia Antipolis, CNRS, Virtual Biology Lab, Parc Valrose, 06108 Nice, France; 4 Institut de Biologia Evolutiva (UPF-CSIC), CEXS-UPF-PRBB, C/Dr Aiguader, 88, Barcelona 08003, Spain

## Abstract

**Background:**

Brain-expressed genes that were created in primate lineage represent obvious candidates to investigate molecular mechanisms that contributed to neural reorganization and emergence of new behavioural functions in *Homo sapiens*. *PMCHL1 *arose from retroposition of a pro-melanin-concentrating hormone (*PMCH*) antisense mRNA on the ancestral human chromosome 5p14 when platyrrhines and catarrhines diverged. Mutations before divergence of hylobatidae led to creation of new exons and finally *PMCHL1 *duplicated in an ancestor of hominids to generate *PMCHL2 *at the human chromosome 5q13. A complex pattern of spliced and unspliced *PMCHL *RNAs were found in human brain and testis.

**Results:**

Several novel spliced *PMCHL *transcripts have been characterized in human testis and fetal brain, identifying an additional exon and novel splice sites. Sequencing of *PMCHL *genes in several non-human primates allowed to carry out phylogenetic analyses revealing that the initial retroposition event took place within an intron of the *brain cadherin *(*CDH12*) gene, soon after platyrrhine/catarrhine divergence, i.e. 30–35 Mya, and was concomitant with the insertion of an AluSg element. Sequence analysis of the spliced *PMCHL *transcripts identified only short ORFs of less than 300 bp, with low (VMCH-p8 and protein variants) or no evolutionary conservation. Western blot analyses of human and macaque tissues expressing *PMCHL *RNA failed to reveal any protein corresponding to VMCH-p8 and protein variants encoded by spliced transcripts.

**Conclusion:**

Our present results improve our knowledge of the gene structure and the evolutionary history of the primate-specific chimeric *PMCHL *genes. These genes produce multiple spliced transcripts, bearing short, non-conserved and apparently non-translated ORFs that may function as mRNA-like non-coding RNAs.

## Background

There is an ancient [1] but still active debate in the molecular biologist community about the relative contribution of structural genomic modifications [2] that could account for the phenotypic differences observed between primate species, particularly in the emergence of new brain structure and functions [3,4]. Very debated results were found when determining the Ka/Ks ratio, a tentative indicator of positive Darwinian selection, in the coding region of genes expressed in the mammalian brain [5-7]. However, genome-wide comparative studies of mammalian promoters suggested an accelerated evolution of primate promoters during the last 25 million years [8-10]. Recently, divergence between human and chimpanzee sequences have been re-evaluated to almost 5%, resulting mainly from indel events [2,11-13] and copy number variants (CNVs) that strongly contributed in shaping primate genomes [14], offering therefore a wide variety of sites at which primate lineage-specific genetic novelty could happen. Indeed, recent segmental duplications are particularly enriched in genes that display expression differences between humans and chimpanzees [15]. In addition, an accelerated rate of contraction or expansion in gene families, including brain-expressed genes, operated in primates when compared with other mammals [16]. On a larger scale, CNVs contributed significantly to diverse expression phenotypes in primates [17] and to emergence of complex or sporadic diseases in humans [18]. To reconcile apparently conflicting data, we previously proposed that, in parallel to single nucleotide mutations that confer alterations in the gene expression patterns or amino acids sequences, genomic rearrangements may have played an important role during primate evolution, providing creation of novel but rare regulatory modules as well as protein coding and/or non-coding genes [19-21]. Indeed, combination of exon shuffling, retrotransposition and gene promoter fusion have led to genes harbouring completely new structures and expression patterns selectively in the primate lineage (reviewed in [22,23]). These rare events would have been nevertheless particularly important in shaping human genes found expressed in reproductive organs, as exemplified by the chimeric *POTE-actin *genes [24], or involved in hominoid brain neurotransmission, as exemplified by the *GLUD2 *gene [25].

The study of primate-specific gene creation and early evolution requires the discovery of genes that have retained characteristic features of their youth [26]. The *PMCHL *system, which combines the retroposition/exon shuffling and the segmental duplication models, has been one of the first hominoid-specific gene creation model described [19-21,27,28]. We have shown that these genes have been created in the hominoid lineage through i) retroposition at the ancestral chromosome 5p14 locus in catarrhini of an antisense *pro-melanin-concentrating hormone *(*PMCH*) gene transcript, ii) local rearrangement leading to a truncated version of the retrogene, iii) sequence remodelling (indel and mutation accumulation that allowed creation of exons) and iv) final duplication at the ancestral 5q13 locus in hominids. Furthermore, processed and unprocessed transcripts were characterized in a human fetal brain library [19] as well as in developing human brain [27]. These mRNAs were found to encode a putative nuclear protein of 8 kD, named VMCH-p8, that was only identified using *in vitro *translation systems or transfected cell models [27]. However, many questions remained unsolved regarding the region and time of insertion of the retrogene, the fine structure of both genes (complete exon/intron structure), their expression patterns (in particular the relative abundance and tissue-specificity of processed transcripts) and their protein coding potential in human cells.

In this paper, we established the structure of *PMCHL1/PMCHL2 *genes and demonstrated that alternatively spliced transcripts encompassing exons 1 to 6 are mostly expressed in human testis. We established further the evolutionary history and regional organization of *PMCHL *genes at both loci on human chromosome 5 and proposed that a single retroposition event followed by point mutations provided novel exonic sequences in transcriptional sense direction. Several short open reading frames (ORFs) were found encoded within the spliced *PMCHL *RNAs but most of them were not conserved in the primate lineage. This suggests a lack of overt functionality of these ORFs, even though the existence of a small species-specific protein cannot be ruled out. Finally, we attempted to identify proteins generated from *PMCHL *genes in macaque and human tissues with an antiserum directed against VMCH-p8 and its variants but we failed to detect them. Therefore, spliced transcripts from the primate-specific *PMCHL1/PMCHL2 *genes would likely represent mRNA-like non-protein coding RNAs (ncRNAs).

## Results and discussion

### 1. Structure and distribution of spliced *PMCHL *transcripts in human brain and testis

**Figure 1:**
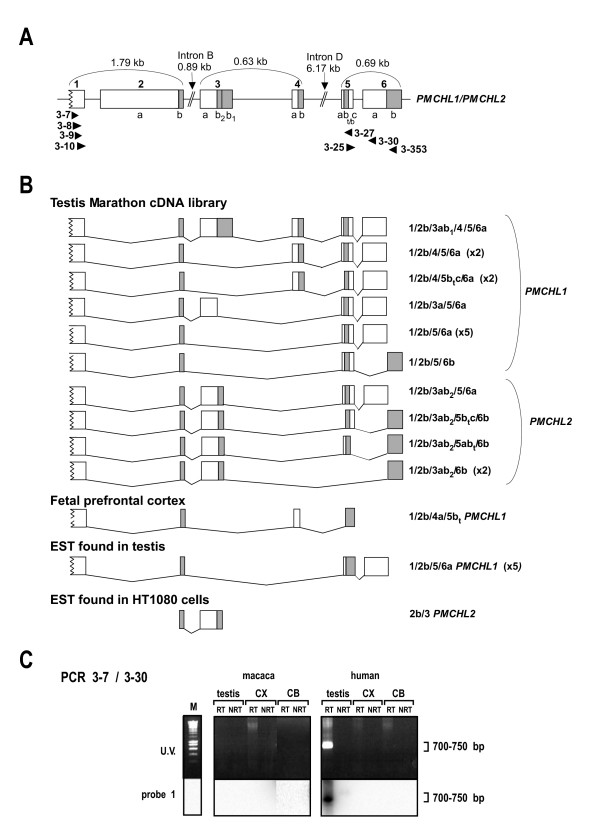
**Identification of spliced *PMCHL *transcripts**. (A) *PMCHL *exon/intron structure as deduced from previous [19] and present transcript sequences. Primer positions are indicated. (B) Identification of alternative spliced transcripts by PCR and sequencing. For human testis, transcripts harbouring exons 1 and 6a were amplified using primer pair 3–9/3–30, and transcripts harbouring exons 1 and 6b were amplified using primer pair 3–9/3–353, followed by 3–10/3–353. For human fetal brain, PCR primer pairs were 3–7/3–30, followed by 3–8/3–27. Documented ESTs [GenBank:AI203691; EMBL:BX091674; GenBank:AA724728; GenBank:BG184695] corresponding to spliced transcripts are represented. The number of independent clones for each transcript identified in our previous [19] and present studies are indicated in brackets. The 3b splice donor site differs in *PMCHL1 *(named 3b_1_) and *PMCHL2 *(named 3b_2_). Two 5b splice acceptor sites (indicated by t/b), separated by four nucleotides, were identified. GenBank accession numbers of transcripts are: [GenBank:EU921424, GenBank:EU921425, GenBank:EU921426, GenBank:EU921427, GenBank:EU921428, GenBank:EU921429, GenBank:EU921430, GenBank:EU921431, GenBank:EU921432, GenBank:EU921433, GenBank:EU921434, GenBank:EU921435, GenBank:EU938381]. (C) RT-PCR and Southern blot analysis of spliced transcripts in human and macaque adult testis, prefrontal cortex (CX) and cerebellum (CB). Spliced transcripts were detected only in human testis. PCR amplification was with primer pair 3–7/3–30. Molecular weights are indicated. M, size markers; RT, reverse transcribed; NRT, non-reverse transcribed.

In our previous study [19], we characterized several alternatively spliced transcripts harbouring exons 1 to 5 of the original *PMCHL1/PMCHL2 *genes. Six transcripts corresponding to *PMCHL1 *spliced RNA were found in testis and/or fetal brain. Two *PMCHL2 *spliced RNA were reported only in testis. In order to further precise the exon/intron structure of *PMCHL *genes and to further investigate the tissue distribution of spliced *PMCHL *transcripts, we examined the presence of additional RNAs in human testis and cortex Marathon cDNA libraries, as well as in a human fetal prefrontal cortex sample. For this, we performed PCR experiments (Figure 1) using primer pairs designed to amplify (in one or two rounds of PCR) transcripts encompassing the most distant known exons, previously named exons 1 and 5. Sub-cloning of the PCR products and sequencing of individual clones allowed the discovery of a novel exon, located between exon 2 and former exon 3. It was named exon 3 and previously named exons 3, 4 and 5 are now renamed exons 4, 5 and 6 (Figure 1A, top panel).

We identified six *PMCHL1 *spliced variants in adult testicular Marathon cDNA library (Figure 1B), two of which [GenBank:EU921424, GenBank:EU921428] had already been identified in the same cDNA library [GenBank:AY008408, GenBank:AY008410, respectively]. However, transcript [GenBank:EU921424] bears one A to G mutation within exon 1, resulting in an arginine to glycine mutation in ORF1. Noteworthy, transcript [GenBank:EU921428] corresponds also to two testis ESTs (IMAGE clone 1753807 [GenBank:AI203691; EMBL:BX091674] and IMAGE clone 1326573 [GenBank:AA724728]), and was also found in a human fetal brain Marathon cDNA library in our previous study [19]. This apparently abundant transcript harbours exons 1-2b and 6a, like most spliced transcripts in testis which were obtained in a single round of PCR. In contrast, transcripts harbouring exon 6b were identified after two rounds of PCR and never contained exon 6a. Transcripts containing exon 2a were never observed in the present study.

**Figure 2:**
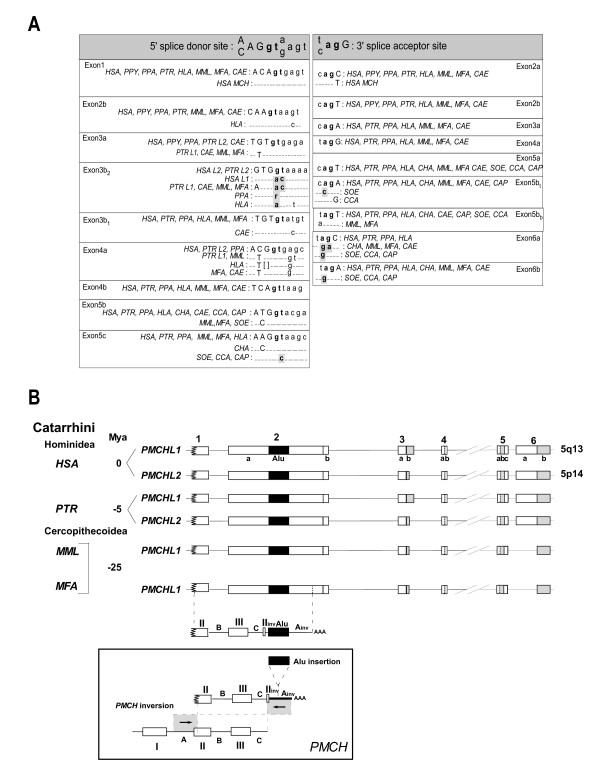
**Phylogenetic analysis of the intron-exon boundaries of the *PMCHL *genes**.(A) Comparative sequence analysis of *PMCHL *splice donor and acceptor sites. Exonic nucleotides are in uppercase letters, and intronic nucleotides are in lowercase. Consensus splice donor and acceptor sequences are indicated. The most invariant dinucleotides gt and ag are in bold characters. Sequence differences in these dinucleotides are boxed in grey. Dashes indicate identity with the sequence in the first row. Brackets indicate a gap. GenBank accession numbers and positions in http://www.ensembl.org are as follows: *PMCH HSA *[GenBank:NM002674], *PMCHL1 HSA *chr 5p14, 22178218–22188421, *PMCHL2 HSA *chr 5q13, 70707368–70717576, *PMCHL1 PTR *chr 5, 93832488–93866206, *PMCHL2 PTR *chr 5, 44567880–44600471, *PMCHL PPA *[GenBank:EF043264], *PMCHL PPY *[GenBank:AY008415, GenBank:AY008422, GenBank:AY008419], *PMCHL HLA *[GenBank:EF043266], *PMCHL CAE *[GenBank:EF043268], *PMCHL MML *chr 6, 22205153–22215832, *PMCHL MFA *[GenBank:EF043267], *PMCHL CHA *[GenBank:AY008430], *PMCHL SOE *[GenBank:EF043262], *PMCHL CAP *[GenBank:EF043263], *PMCHL CCA *[GenBank:AY008431]. (B) Schematic representation of the *PMCHL1 *and *PMCHL2 *exon/intron structures in *Homo sapiens *(*HSA*), *Pan troglodytes *(*PTR*), *Macaca mulatta *(*MML*) and *Macaca fascicularis *(*MFA*). The position of the retroposed sequence derived from the antisense strand of the *PMCH *locus is indicated and detailed correspondence between the sequences is given (inset). Times of divergence are indicated (Mya).

We also identified four novel alternative *PMCHL2 *splicings in adult testis, which all contained a partial exon 3 (3b_2_) shorter than the original exon 3 (3b_1_) observed in a *PMCHL1 *transcript (Figure 1B). Sequence analysis (see Figure 2) revealed that mutations in the *PMCHL2 *sequence created a novel gene-specific splice donor site (3b_2_) which is systematically used in the present *PMCHL2 *transcripts.

We further identified one novel *PMCHL1 *splice variant in a fetal prefrontal cortex sample (Figure 1B). This transcript was the only one to harbour an alternative splice donor in exon 4, which was never observed in testis RNAs.

In our previous study [19], we reported two alternative acceptor sites in exon 5b (previously named exon 4b) separated by only four nucleotides and with apparent testis- and brain-specificities (indicated by superscript t/b in Figure 1). Our present results show that most testis transcripts use the exon 5a splice acceptor site, and two use the alternative 5b_t _site. However, the *PMCHL1 *transcripts identified in fetal brain also use the alternative 5b_t _site indicating that it could not be considered anymore as a testis-specific splice acceptor site. The alternative 5b_b _site, previously reported in a fetal brain transcript was not found in our present study.

In contrast to human testis and fetal brain, we could not detect any spliced *PMCHL *RNA harbouring exons 1 and 6a in the human adult cortex Marathon cDNA library using the 3–7/3–30 primer pair. This primer pair was further used in RT-PCR experiments combined with Southern blot to determine the tissue distribution of the spliced transcripts in testis, prefrontal cortex and cerebellum in adult human and macaque (Figure 1C). In agreement with our results using the Marathon cDNA libraries, we detected spliced *PMCHL *RNA harbouring exons 1 and 6a in human adult testis, but not in adult prefrontal cortex and cerebellum. Thus, *PMCHL1 *transcripts are found in testis and fetal brain and are more abundant than *PMCHL2 *transcripts that are observed only in testis. In addition, *PMCHL2 *gene expression was reported in HT1080 cells subjected to RAGE (random activation of gene expression), in which an EST [GenBank:BG184695] encompassing exons 2a and 3 of *PMCHL2 *has been identified.

In macaque, no spliced transcripts were identified by Southern blot (Figure 1C) in agreement with our sequence analysis indicating that the macaque *PMCHL1 *gene lacks the exon 6a acceptor splice site (see below).

Taken together, our findings indicate that *PMCHL1 *and *PMCHL2 *genes: i) give rise to a complex pattern of alternative splicings, ii) are subject to distinct tissue-specific expressions and iii) are developmentally regulated (i.e. expressed in fetal but not adult cortex).

The finding that a rather high diversity of spliced transcripts are present in testis is not surprising, because of the permissive chromatin environment present in gonads, allowing high transcriptional activity even from weak tissue-specific promoters [29]. Thus, most retroposons evolve into non-functional pseudogenes that are transcribed only in the testis [30]. However, the abrupt emergence of a new chimeric gene in primates could potentially contribute to reproductive barriers and thus play a role in speciation [31]. In this regard the hominoid-specific oncogene *Tre2 *appears expressed only in testis while the two parental genes *USP32 *and *TBC1D3*, that fused to generate the *Tre2 *gene, are expressed in a broad range of human tissues [32]. In addition, the presence of spliced *PMCHL *transcripts in fetal brain, is rather suggestive of a functional role during human brain development. This would imply that the retroposon acquired an active promoter and has been subjected to selection pressure. Whether these spliced *PMCHL *transcripts actually play a functional role in testis and fetal brain is an obvious question, which we further addressed below.

### 2. Evolutionary history of the *PMCHL *genes

#### Retroposition-driven creation of PMCHL1 gene occurred 30–35 Mya in primate lineage

To gain further insights into the evolutionary history of the *PMCHL *gene family, we extended our previously initiated sequence analysis of *PMCHL *and *PMCH *genes. For this, genomic DNAs were PCR amplified with the *PMCHL*- or *PMCH*-specific primer pairs indicated in Table 1 and the PCR products were sequenced. Novel sequences were submitted to GenBank under the following accession numbers. For *PMCHL1*: [GenBank:EF043262] (*SOE*), [GenBank:EF043263] (*CAP*), [GenBank:EF043264] (*PPA*), [GenBank:EF043265] (*PTR*), [GenBank:EF043266] (*HLA*), [GenBank:EF043267] (*MFA*), [GenBank:EF043268] (*CAE*). For *PMCH*: [GenBank:EU916242] (*TSY*), [GenBank:EU916243] (*MFA*), [GenBank:EU916244] (*CAE*), [GenBank:EU916245] (*SOE*), [GenBank:EU916246] (*CCA*), [GenBank:EU916247] (*PPA*), [GenBank:EU916248] (*PTR*), [GenBank:EU916249] (*HLA*)].

**Table 1:** Sequences of oligonucleotides used for PCR

*forward primer*	*sequence*	*reverse primer*	*sequence*	*T°*	*gene*
*Sequencing*					
1–2	CTCAAGGTATTTTACTTTCAGCATCC	1–3	TGCAGAATTTTCACAAAGTTTAATGCAC	56°C	*PMCH*
3–4	GGCCATAGGGTGGTTTGG	3–19	TGAGTAGATAAAAGGACTGACTT	56°C	*PMCHL*
3–18	AAGTCAGTCCTTTTATCTACTCA	3–22	TAACCTTGCTTTCTTCCTTTCTATA	57°C	*PMCHL*
3–15	GCGTCAGTGTCCTAATGCAT	3–68	GAATTTCTGAGCTGTGTTGTGC	58°C	*PMCHL*
3–18	AAGTCAGTCCTTTTATCTACTCA	3–27	TGATAACGTGAAATCGTACCAT	54°C	*PMCHL*
3–20	ACTCCACGTCAAGACAGTTGCA	3–30	GATGGAGGTAAACCAAGGAGG	60°C	*PMCHL*
3–72	TTCTGGTTTTCACAGTAACTGATCT	3–142	GGGAAATCAGTGAGTGGAGTAGGAA	58°C	*PMCHL*
3–110	GCTGAAATCCTTCCTCAAGA	3–33	AGCTGATATCCTAGAAGTAG	53°C	*PMCHL*
					
*PCR*					
3–9	CTGAGAATGGGGTTCAGGATAC	3–30	GATGGAGGTAAACCAAGGAGG	58°C	*PMCHL*
3–9	CTGAGAATGGGGTTCAGGATAC	3–353	TTTAACAATTGAACACATGTAATCATT	53°C	*PMCHL*
3–10	TGGGGATGAAGAAAACTCAGCTAA	3–353	TTTAACAATTGAACACATGTAATCATT	53°C	*PMCHL*
3–7	CAATGGGATTATGCTGTCACAA	3–30	GATGGAGGTAAACCAAGGAGG	58°C	*PMCHL*
3–8	AACATAATTTCTTAAATCATGG	3–27	TGATAACGTGAAATCGTACCAT	56°C	*PMCHL*
					
*Probe 1*					
3–25	TGAGATGTAAAGAGACCACCTT	3–30	GATGGAGGTAAACCAAGGAGG	58°C	*PMCHL*

Annealing temperature (T°) that was used for PCR and gene-specificity are indicated for each oligonucleotide pair.

We first carried out the phylogenetic analysis of the exon/intron boundaries of the *PMCHL *genes (Figure 2A, B). Consensus gt/ag splice donor and acceptor sites are present in all species bearing the retroposon, i.e. all catarrhines of this study, with the exception of the splice acceptor site of exon 6a which is present only in hominoids. Moreover, the exon 5c donor site and the 6a and 6b splice acceptor sites were not consensual in the platyrrhines analysed here, i. e. before the retroposition event, suggesting that canonical splicing could not occur in this ancestral region before the arrival of the retroposed sequence. Our sequence analysis showed that these splice sites were created *de novo *through single nucleotide mutations. Thus, the splicing between exons 5 and 6 corresponds to *de novo *exonisation and not to an Alu-driven exonisation mechanism [33], exon 6 being absent in platyrrhines and then conserved through selection pressure. In contrast, splice sites of exons 3 and 4 pre-dated the retroposition event, indicating that a fusion of the *PMCH*-derived exons with pre-existing exons is likely to have occurred. Whether these exons were expressed before the retroposition remains to be determined, but no expressed sequence tag corresponding to exons 3 and 4 alone could be identified in mammalian EST databases. Furthermore, chimeric transcripts formed by transcription of two consecutive genes into a single RNA can occur quite frequently in human cells [34]. A similar mechanism may be involved in the production of fused transcripts encompassing either exon 3 or 4 of *PMCHL1/PMCHL2 *genes. However, further characterization of putative promoters inside the *PMCHL *genes needs to be done before involving such RNA domain accretion process on regulation of these genes.

**Figure 3:**
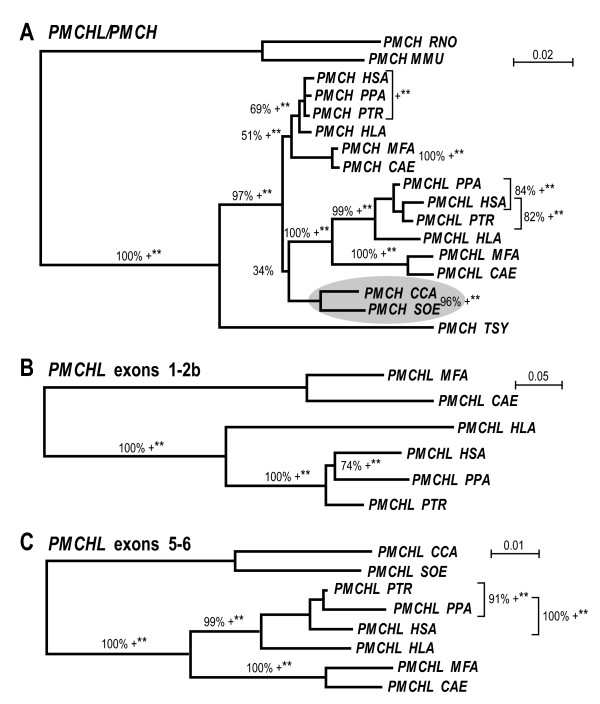
**Phylogenetic analyses of the *PMCH *and *PMCHL *genes. **Phylogenetic analyses of (A) the alignable *PMCH *and *PMCHL *sequences, of (B) the region encompassing exon 1 to exon 2b of *PMCHL*, and of (C) the region encompassing exon 5 to exon 6 of *PMCHL*. All three unrooted trees were obtained using a neighbour-joining (NJ) method. Branches also found by maximum likelihood (G option) and parsimony are indicated with ** (p < 0.01) and +, respectively. Grey oval indicates unresolved localization of the platyrrhine *PMCH *branch. *Mus musculus (MMU*) and *Rattus norvegicus (RNO) *are used as outgroups in (A). GenBank accession numbers and positions in http://www.ensembl.org are as indicated in Figure 2, and the following: *PMCH PPA *[GenBank:EU916247], *PMCH PTR *[GenBank:EU916248], *PMCH HLA *[GenBank:EU916249], *PMCH MFA *[GenBank:EU916243], *PMCH CAE *[GenBank:EU916244], *PMCH CCA *[GenBank:EU916246], *PMCH SOE *[GenBank:EU916245], *PMCH TSY *[GenBank:EU916242], *PMCH RNO *[GenBank:NM012625], *PMCH MMU *[GenBank:NT039500], *PMCHL1 HSA *[GenBank:AY028318, GenBank: AY028319], *PMCHL2 HSA *[GenBank:AY028320, GenBank:AY028321], *PMCHL1 PTR *[GenBank:EF043265].

A nucleotidic phylogenetic analysis (Figure 3) was performed to date more precisely the retroposition event. As *PMCHL *genes encompass part of the *PMCH *gene sequence (sense and antisense), it was possible to align primate sequences of these specific parts of the *PMCHL1 *and *PMCH *genes. The phylogenetic analysis was performed using the parsimony, maximum-likelihood and neighbour-joining methods and with the rat and mouse *PMCH *sequences as outgroups (Figure 3A). All phylogenetic methods led to congruent data, with high bootstrap values with the neighbour joining method. The tree showed an apparent aberration relative to our present knowledge concerning the relationship between species and the creation of the *PMCHL1 *gene (circled in Figure 3A). We expected the *PMCH *sequences of *Cebus capucinus *and *Saguinus oedipus *to be grouped with the *PMCH *sequence of *Tarsius syrichta *rather than with the *PMCHL *sequences. However, a noteworthy low bootstrap value (34%) was found for this branching. The position of the *PMCHL *sequences as well as the uncertainty for positioning the *Cebus capucinus *and *Saguinus oedipus PMCH *sequences suggest that the retroposition event leading to the *PMCHL1 *gene occurred very shortly (likely within 5 million years) after the split of platyrrhini/cathyrrhini, i.e. 30–35 Mya [35,36].

When focusing on the *PMCHL *genes, in the regions encompassing exons 1-2b (Figure 3B) and exons 5–6 (Figure 3C) the nucleotidic phylogenetic trees fully correspond to accepted species trees, indicating that no particular and global (since here the entire gene sequences were used) evolutionary event interfered. However, we observed a difference in the *Pan troglodytes*/*Pan paniscus*/*Homo sapiens *positioning between the two phylogenetic trees. This simply corresponds to an inherent irresolution in the hominidae speciation, which could be inferred (or not) from a complex speciation with interbreeding before final separation of chimpanzees, gorillas and humans [37-39]. All three species should probably be grouped under the *Pan *or *Homo *clade as previously suggested [35,40,41].

We previously reported the presence of a complete Alu-Sq sequence element within *PMCHL *exon 2 [27], but the insertion event into *PMCHL1 *could not be dated precisely. It is worth noting that this sequence appears to be an AluSg, and not an Alu Sq element. Our present sequence analysis reveals that all primate species carrying the *PMCHL1 *exon 2 harbour the AluSg sequence (Figure 2B). Thus, the insertion was likely concomitant to the retroposition, occurring after the divergence of *Cebus *species (*C. apella *and *C. capucinus*), and before the divergence of the cercopithecoids, approximately between 30–35 Mya.

#### A 92 kb element encompassing PMCHL1 and adjacent intronic/exonic sequence of CDH12 on 5p14 duplicated to create PMCHL2 on 5q13 at the time of hominid divergence

**Figure 4:**
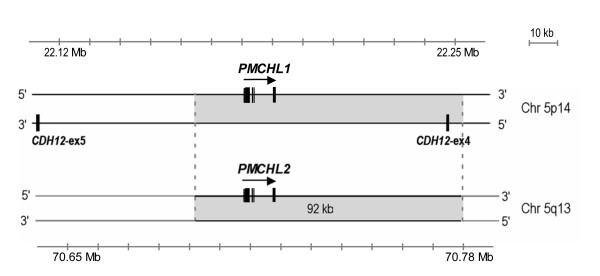
**Schematic map of *PMCHL *gene positions at human chromosome 5p14 and 5q13 loci**. The six exons of *PMCHL1 *locate within 10 kb on chromosome 5p14, on the opposite strand of *CDH12 *intron 4. *PMCHL2 *arose by duplication of a 92 kb fragment (in grey) encompassing *PMCHL1 *onto chromosome 5q13. Positions according to http://www.ensembl.org nomenclature are indicated.

We previously proposed [19] that *PMCHL2 *was created from a duplication of a large, but undefined in size, genomic DNA fragment comprising *PMCHL1*, "jumping" from ancestral hominid chromosome 5p14 to 5q13. Here, we precisely determined the limits of the duplicon by similarity using a BLAST search [42]. It appeared that a fragment of 92 kb, encompassing 17 kb upstream and 65 kb downstream of the 10 kb of *PMCHL1 *was duplicated (Figure 4). This 92 kb duplicon corresponds to a large part (88 kb) of the 5' portion of intron 4 of the *CDH12 *gene encoding brain cadherin (as defined in http://www.ensembl.org), as well as *CDH12 *exon 4 and the last 4 kb of its intron 3. No other exons (found elsewhere in the genome) than those derived from the *PMCH *gene were found in the duplicon. The percentages of identity between the 5p and 5q elements were equivalent all along the duplicon (i.e. in the *PMCHL *genes, and in the 5' and 3' flanking regions), and are close to 98%, in agreement with a very recent duplication event.

We have therefore precisely mapped the limits of the *PMCHL1/PMCHL2 *duplicon. A question concerning the creation of novel genes, particularly in the case of segmental duplication, is relative to the acceptor sites. Which kind of sequences allows invasion by foreign sequences? Which kind of sequences can we find at the segmental duplication boundaries? No clear boundaries specificities are described for segmental duplication except for a significant enrichment in short interspersed elements (SINEs) such as young Alu Y and Alu S sequences and other repeats similar to these involved in Ig heavy chain recombination in pericentromeric and interstitial segmental duplications [2,13,43]. Alu mediated DNA duplications have exceptionally been reported in eukaryotes [44]. These duplications appeared however to affect mainly hyper-recombinogenic chromosomal regions, and particularly for secondary duplications [43]. Long interspersed elements (LINEs) like Line 1 elements were also directly (i.e. not only favouring Alu sequences duplication) implicated in exon recombination and have been proposed to mediate exon shuffling [22], but none of the previously described human chimeric genes [45] harbour this kind of element at its boundaries. In the case of *PMCHL1/L2*, no particular SINEs or LINEs sequences could be found at the boundaries, neither at the first insertion site of the *PMCH *antisense retroposon (in an intron of the *Brain Cadherin *(*CDH12*) gene at the 5p14 locus), nor at the 5q13 locus when creating *PMCHL2*. Recently, a duplication-driven model for DNA transposition has been put forth by Eichler's group [46] suggesting that the probability for a DNA element to be duplicated correlated with the degree of proximity to so-called core duplicons. In this context, a core duplicon named Glu^5–10 ^and corresponding to a truncated version of the *GUSB *gene, has been found in close vicinity to *PMCHL1 *and *PMCHL2 *genes on 5p14 and 5q13 respectively [20]. Whether the proximity with this duplicon was determinant for emergence of the *PMCHL2 *gene remains at this stage a matter of speculation but the timing of both Glu^5–10 ^duplicon expansion and intrachromosomal duplication of *PMCHL1 *in primates fits very well. These duplication events are also congruent with the global surge in intrachromosomal duplications at the time of hominid divergence, as previously predicted [21] and experimentally proven [47].

### 3. Analysis of the protein coding potential of *PMCHL *transcripts

**Figure 5:**
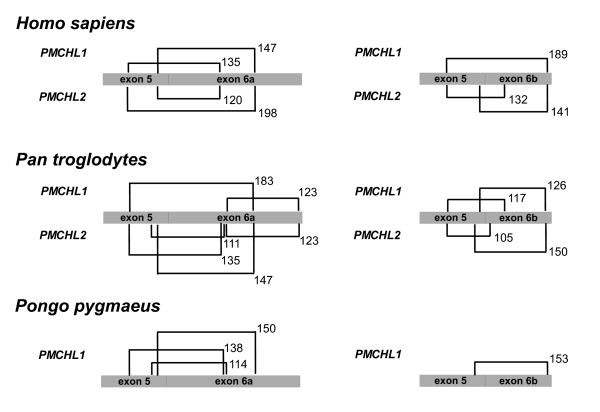
**Mapping of exon 5-6a and exon 5-6b ORFs**. Schematic representations of ORFs found on human *PMCHL1 *and *PMCHL2 *transcripts harbouring exons 5-6a or 5-6b, and on the corresponding putative transcripts in *Pan troglodytes *and in *Pongo pygmaeus*. ORFs > 100 bp are represented by brackets at their relative positions. ORFs are not at scale due to sequence insertions/deletions, but ORF lengths (in base pairs) are indicated.

We next addressed the protein coding potential of the spliced *PMCHL *RNAs. We examined the sequences of all *PMCHL *transcripts reported in the present and in our previous study [19] to identify ORFs longer than 100 bp. Ten short ORFs of less than 300 bp were found. *PMCHL *transcripts harbouring exons 5-6a or 5-6b present all together seven ORFs that are 120 to 198 bp in length (Figure 5), i.e. they would encode proteins of 40 to 66 amino acids. ORFs of less than 300 bp (i.e. 100 amino acids) are often assumed not to be translated. However, many well known functional proteins of less than 100 amino acids in length have been reported, including the small inducible cytokine families CCL and CXCL [48], and the xenobiotic defensin and defensin-related cryptidin factors [49]. Furthermore, a recent study has shown that among the 31,035 predicted proteins encoded by the 102,801 FANTOM mouse full-length cDNA sequences, 12% of the proteins (i.e. 1,683 proteins) are less than 100 amino acids in length [50]. This suggests that there might be up to 4 times more small proteins than the 424 entries present for *Mus musculus *to date in the SwissProt protein database (release 56). Interestingly, most of the small proteins with known function are evolutionarily conserved [48] or present conserved sequence motifs [49]. Notably, a recent report [51] indicates that ORFs < 300 bp in length, that are not evolutionarily conserved, are unlikely to be translated into functional proteins. Given that the *PMCHL *ORFs present on exons 5-6a and 5-6b are not conserved among *Homo sapiens*, *Pan troglodytes *and *Pongo pygmaeus*, due to frameshift-causing insertions/deletions (Figure 5), we propose that these ORFs are most likely non-functional.

The longest ORF identified on *PMCHL *transcripts is 294 bp (98 amino acids) long and locates within the Alu sequence in exon 2a. Four transcripts harbouring exon 2a were identified in testis in our previous study [19]. However, the corresponding putative protein is not conserved due to a single nucleotide insertion in the human *PMCHL1 *sequence causing a frameshift in the middle of the ORF. Therefore, this ORF is also spurious according to the criteria of Clamp and colleagues [51].

**Figure 6:**
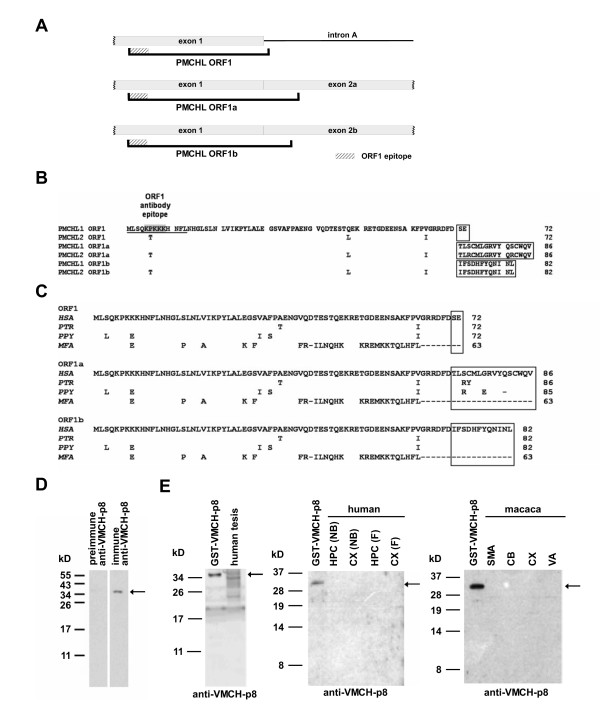
**Analysis of the coding potential of VMCH-p8 and its variants in human and macaque tissues**. (A) Schematic representation of *PMCHL *ORF1, encoding the putative VMCH-p8 protein, present on unspliced RNA, and its variants, ORF1a and ORF1b, present on spliced RNAs. (B) Sequence comparison of *PMCHL1 *and *PMCHL2 *ORF1, ORF1a and ORF1b. The ORF1 epitope is underlined. The putative nuclear localisation signal (NLS) is shaded in grey. The variable C-terminal parts are boxed. (C) Sequence comparisons of *PMCHL1 *ORF1, ORF1a and ORF1b in *Homo sapiens *(*HSA*), *Pan troglodytes *(*PTR*), *Pongo pygmaeus *(*PPY*) and *Macaca fascicularis *(*MFA*). The variable C-terminal parts are boxed. (D) Specificity of the anti-VMCH-p8 antiserum. In Western blots, immune anti-VMCH-p8 serum detects the recombinant GST-VMCH-p8 protein (12.5 ng) migrating at 34 kD (arrow). (E) Western blot analysis of the expression of VMCH-p8 and its variants *in vivo *in human and macaque tissues. GST-VMCH-p8 (12.5 ng), human adult testis proteins (25 μg), new-born (NB) and fetal (F) hippocampus (HPC) and prefrontal cortex proteins (CX) (80 μg each), and macaque supplementary motor area (SMA), cerebellum (CB), prefrontal cortex (CX) and visual area (VA) proteins (80 μg each) were analysed using the anti-VMCH-p8 antiserum. The arrow points to GST-VMCH-p8 migrating at 34 kD.

*PMCHL *transcripts encompassing exons 1-2a and 1-2b harbour two ORFs, named ORF1a and ORF1b, respectively (Figure 6A, B, C). Even though these ORFs are also less than 300 bp in length, they present the same lengths in *Homo sapiens*, *Pan troglodytes *and *Pongo pygmaeus*, and share > 90% sequence identity. In *Macaca fascicularis*, the ORF is shortened and runs only on exon 1 due to the presence of an early stop cordon. These ORFs are of particular interest because they share a large part of sequence identity with ORF1 present on unspliced *PMCHL *RNA, and with the pro-MCH precursor because it mainly locates in exon 1, i.e. in the region derived from exon 2 of the ancestor *PMCH *gene. The putative 8 kD protein corresponding to ORF1, previously named VMCH-p8, presents a putative nuclear localisation signal (NLS) at the N-terminus (KPKKK, shaded in grey in Figure 6B), and is among the longest ORFs, encoding 72 amino acids (Figure 6A, B, C). In a previous study [27], we examined the protein coding potential of ORF1 carrying out *in vitro *translation experiments and COS-7 cell transfections with the Flag epitope-tagged ORF1. The results indicated a weak protein-coding potential, depending on particular plasmid constructions, providing mRNA stabilising elements and enhanced promoter activity [27]. In the present study, we used a VMCH-p8 antiserum directed against the thirteen N-terminal VMCH-p8 amino acids, comprising the putative NLS (see Figure 6B). This allows the determination of the expression of ORF1, as well as the ORF1a and ORF1b variants (sharing the N-terminal epitope). The reactivity of the VMCH-p8 antiserum was demonstrated in Western blot experiments using a recombinant GST-VMCH-p8 protein produced in bacteria. VMCH-p8 antiserum recognized the GST-VMCH-p8 protein, migrating at about 34 kD, whereas the preimmune serum did not (Figure 6D). Next, we used the VMCH-p8 antiserum to examine expression of ORF1 and its variants in human and macaque tissues (Figure 6E). We tested human adult testis, hippocampus and prefrontal cortex extracts from a new-born and a foetus, as well as four *Macaca fascicularis *cerebral areas (supplementary motor area, cerebellum, prefrontal cortex and visual area). These tissues and cerebral areas were chosen for the presence of ORF1-bearing *PMCHL *transcripts in RT-PCR experiments ([27,28]; our unpublished data). In our Western blot experiments, no signal could be detected in all human and macaque tissues tested, at the expected size of 8–9 kD for the putative VMCH-p8 protein and its variants. This strongly suggests that these putative proteins are not translated *in vivo *in the human and macaque tissues that we tested. We further carried out Western blot and immunoprecipitation experiments on HEK293 cells transfected with *PMCHL1/2 *sequences bearing ORF1 to detect low levels of VMCH-p8 protein. Even though high levels of ORF1-bearing *PMCHL1/2 *transcripts were detected by RT-PCR, no signal corresponding to the VMCH-p8 protein could be detected (data not shown). One explanation for the lack of protein detection, that we cannot exclude, is a very low protein expression level below our detection threshold. Also, for the putative *Macaque *protein, we further cannot exclude an altered epitope-recognition of the antibody due to a lysine to glutamic acid mutation within the epitope. Assuming that the failure to detect the VMCH-p8 protein or its variants is not due to these technical limitations, the lack of translation of ORF1 like-bearing mRNAs could reside in the moderate consensus with the optimal sequence for translation initiation described by Kozak [52]. Actually, only the consensual adenine at position -3 is present.

What might be the role(s) of the large variety of spliced *PMCHL *mRNAs in human testis and fetal brain? It is tempting to propose that these *PMCHL *transcripts work mainly as an mRNA-like non-protein coding RNA (ncRNA). Since the realization that 98% of the transcriptional output in mammals consists of ncRNAs, the enthusiasm for this class of RNAs has grown tremendously [53-55] and has been granted its own NONCODE database [56]. Numerous classes of ncRNAs have been reported, most of which are small ncRNAs (including miRNAs, siRNAs and snoRNAs), but also long ncRNAs (ranging from 1 to more than 100kb) such as *Xist *and the antisense *Tsix *transcripts involved in × inactivation in mammals [57,58], or the *Air *RNA that appears to be responsible for imprinted repression of nearby genes (including *Igf2r *gene) through an antisense-mediated mechanism [59]. Several mRNA-like ncRNAs that are transcribed by RNA polymerase II, spliced and polyadenylated have also been reported [60-62], including in human [63,64]. Interestingly, many small ncRNAs are located in introns of coding or non-coding mRNAs [54,65]. The functional roles of ncRNA are diverse, corresponding mainly to adaptor functions targeting nucleic acids to various enzymatic complexes (involved in RNA processing, splicing, transcription...) and gene expression regulation/silencing (involved in virtually all cellular functions).

Do the *PMCHL *transcripts host small ncRNAs in their introns, and/or do the *PMCHL *transcripts control the expression of neighbouring genes in *cis *(an obvious candidate is the *CDH12 *gene) or in *trans *through RNA-RNA duplexes (obvious candidates are the *PMCH *and *Antisense RNA Overlapping MCH *(*AROM*) genes)? We are now addressing these intriguing questions.

## Conclusion

We provide here new data concerning spliced *PMCHL *transcripts, further precising the *PMCHL *gene structure. Sequencing data of the *PMCHL *genes in several non-human primates offered a substantial improvement of the creation model proposed previously [19]. In particular, we proposed that the initial retroposition occurred within an intron of the *CDH12 *gene soon after platyrrhine/catarrhine divergence and was concomitant with the insertion of an Alu Sg element. Our sequence analysis further points to the presence of short ORFs that present little or no evolutionary conservation, suggesting that spliced *PMCHL *transcripts are non-protein coding RNAs. This proposal is further supported by our expression analysis of the most relevant *PMCHL *ORFs in human and macaque tissues, which failed to detect any corresponding protein.

## Methods

### Tissues

Human prefrontal cortex and cerebellum from adults were provided by the National Neurological Research Specimen Bank (Los Angeles, CA, USA) and by the GIE Neuro-CEB (Hôpital de la Pitié-Salpétrière, Paris, France), which collect tissues with the full authorization of the respective local ethical committees. Human adult testis RNA was purchased from BioChain/Cliniscience (France). Dr. A. Coquerel (CHU Rouen, France) provided human prefrontal cortex and hippocampus tissues from a newborn. Dr. D. Jordan (Faculté de médecine, Lyon, France) provided human prefrontal cortex and hippocampus tissues from a foetus. Collection of human new-born and foetal tissues was according to the french legislation of parental consent and with the approval of local ethical committees. Human adult testis total proteins were purchased from BioChain/Cliniscience (France). Testis, prefrontal cortex, cerebellum, visual area, and supplementary motor area samples from three adult macaques (*Macaca fascicularis*) were obtained from Dr. E. Bezard at the Biothèque Primate/Primatech (CNRS, Bordeaux, France), where tissue collection is carried out in agreement with the European Communities Council Directive of November 24, 1986 (86/609/EEC).

### Genomic DNAs

Genomic DNAs were collected from *Cebus capucinus *(gift from B. Dutrillaux, cytogénétique moléculaire et oncologie, CNRS, Institut Curie, Paris, France), *Tarsius syrichta*, *Saguinus oedipus*, *Cebus apella*, *Chlorocebus aethiops*, *Hylobates lar*, *Pan paniscus *and *Pan troglodytes *(gift from Dr P. Dijan, CEREMOD, Meudon, France) and were already used in previous studies [28]. Other genomic samples were kindly provided by San Diego Zoo/CRES (*Pan paniscus, Gorilla g.g., Pongo pygmaeus, Pongo p.abelii, Hylobates lar, Macaca silenus*) and by Prof A. Blancher (Rangueil Hospital, Toulouse, France) (*Cebus appella, Pan troglodytes*). Genomic DNA was isolated from the occipital cortex of a *Macaca fascicularis *(provided by Dr. E. Bezard (Biothèque Primate/Primatech, CNRS, Bordeaux, France) according to the Blin and Stafford's method [66].

### RNA extraction and reverse-transcription

Total RNAs were extracted from human and macaque tissues according to standard guanidium phenol method [67] and using a FastPrep apparatus (FP220A Thermo instrument, Qbiogene, France). Contaminating genomic DNA was removed from RNA preparation by RQ1 RNase-free DNase treatment (Promega) according to the manufacturer's protocol. cDNAs were synthesized by reverse-transcription (RT) of 2 μg of total DNase-treated RNAs using the SuperScript TM II Reverse Transcriptase (Invitrogen) and oligo dT according to the manufacturer's protocol.

### PCR amplification

Oligonucleotides (list provided in Table 1) were purchased from Eurogentec (Belgium).

For genomic DNA, 100–200 ng were PCR-amplified using the oligonucleotide couples indicated in Table 1 and the LA Taq polymerase (Takara) following the supplier's protocol. Thirty-five cycles of amplification were carried out as follows: 30 s at 94°C (denaturation), 30 s at annealing temperature (indicated in Table 1), 1 to 10 min at 72°C (extension). A final extension step of 7 min at 72°C was performed. PCR products were purified using the NucleoSpin kit (Machery Nagel) and sequenced.

For RT samples and Marathon cDNA libraries, 2 μl were PCR-amplified using the indicated primer pairs and the HotMaster Taq DNA polymerase (Eppendorf) following the supplier's protocol. Thirty-eight cycles of amplification were carried out as follows: 30 s at 94°C (denaturation), 30 s at annealing temperature (indicated in Table 1), 2 min at 65°C (extension). A final extension step of 7 min at 72°C was performed. When necessary, nested PCR was performed with internal primers using 2 μl of a 1:20 dilution of the first round products. PCR-amplified fragments were subcloned into the pGEM-T Easy vector (Promega) and transfected into TOP10 thermocompetent cells (Invitrogen) according to the manufacturer's instructions, followed by plasmid DNA preparation using a Qiaprep Spin Miniprep kit (Qiagen) and sequencing.

### Southern blotting

PCR products obtained with primer pair 3–7/3–30 (thirty-five cycles) were electrophoresed on a 1% agarose gel containing ethidium bromide and were visualized under UV. The gel was then denatured 15 min in 500 mM NaOH, 1.5 M NaCl solution, neutralized 15 min in 500 mM Tris, 1.5 M NaCl, and soaked 5 min in 2 × SCC solution (300 mM NaCl, 30 mM sodium citrate). The DNA was transferred overnight as a gravity-dry blot onto a cellulose membrane (Biodyne B, Pall Corporation, FL, USA). The membrane was prehybridized for 4 h at 65°C in Church solution (500 mM Na_2_HPO_4_, pH 6.8, 5% SDS), hybridized overnight at 65°C in fresh Church solution containing previously prepared ^32^P-labeled *PMCHL*-specific probe 1 corresponding to the fragment amplified with primer pair 3–25/3–30 (see Table 1) at 5.10^5 ^dpm.ml^-1^. ^32^P-labeled probes were prepared using the Prime-a-gene labelling system (Promega) according to the manufacturer's protocol. After hybridization, the membrane was washed twice 15 min in 2 × SSPE and twice 10 min in 1 × SSPE. Hybridized radioactive probes were detected with a Fujifilm phosphoimager (FLA-5100).

### DNA sequencing and alignment

Sequencing of PCR-amplified fragments was carried out on both DNA strands using the Ampli Taq Polymerase FS, the Big Dye Terminator 1.1 sequencing kit (Applera), and a ABI PRISM 3100 sequencer (Perkin Elmer). Sequences obtained from the public databases (EMBL/GenBank/DDBJ) and fragments sequenced by PCR were aligned manually using SEAVIEW [68]. Species are: *Homo sapiens (HSA), Pan paniscus (PPA), Pan troglodytes (PTR), Pongo pygmaeus (PPY), Hylobates lar (HLA), Macaca mulatta (MML), Macaca fascicularis (MFA), Cercopithecus hamlyni (CHA), Chlorocebus aethiops (CAE), Cebus capucinus (CCA), Cebus apella (CAP), Saginus oedipus (SOE), Tarsius syrichta (TSY)*.

### Phylogenetic analysis

Phylogenetic dendrograms were reconstructed according to three different methods: Neighbour Joining (BIONJ), Maximum Likelihood (ML, using the Global option), and Maximum Parsimony (MP). For the Neighbour Joining (NJ) analysis, a distance matrix was calculated by DNADIST according to the Kimura two parameters correction. Bootstraps were done using 1,000 replications, BIONJ and Kimura two parameters correction. BIONJ was according to Gascuel [69], ML and MP were from PHYLIP (Phylogeny Inference Package, version 3.573c, distributed by J. Felsenstein, Department of Genetics, UW, Seattle, WA, USA). Phylogenetic analyses were done excluding domains that were not common to every sequence as well as low complexity domains that could not be properly aligned. The phylogenetic dendrograms were drawn using NJPLOT [70].

### Preparation of proteins

Human and macaque tissues were homogenized in RIPA buffer (20 mM Tris HCl, pH 7.4, 150 mM NaCl, 1% NP-40, 0.5% sodium deoxycholic acid, 0.1% SDS, 2 mM EDTA, protease inhibitor cocktail Complete (Roche)) using a FastPrep apparatus (FP220A Thermo instrument, Qbiogene, France), incubated on ice for 30 min, and centrifuged at 20000 × g for 15 min at 4°C. Proteins in the supernatants were quantitated using a commercial Bradford reagent (BioRad).

### Production of recombinant GST-VMCHp8 protein

*PMCHL1 *ORF1 encoding the putative VMCH-p8 protein was sub-cloned into the *BamH1*/*EcoR1 *sites of the pGEX-3X vector, in frame with GST (Amersham Biosciences). The construct was used to transform thermocompetent Rosetta cells (Novagen) and the recombinant GST-VMCH-p8 protein was produced and purified using glutathione-sepharose (Amersham Biosciences) beads according to the manufacturer's instructions.

### Antibodies

Polyclonal antibodies were raised against the putative VMCH-p8 protein encoded by *PMCHL1 *ORF1. A peptide comprising the thirteen N-terminal amino acids of the sequence (MLSQKPKKKHNFL) was designed by Dr B. Cardinaud (IPMC, Valbonne, France), synthesized and coupled to keyhole limpet haemocyanin (KLH) before rabbit immunization (Genaxis, Nîmes, France). Anti-VMCH-p8 antiserum was used at a final dilution of 1:1,000. Secondary HRP-coupled goat anti-rabbit antibodies (Jackson ImmunoResearch) were used at a 1:10,000 dilution.

### Western blotting

Proteins were separated on 12% Tris-glycine or 16.5% Tris-tricine gels under reducing conditions and transferred to nitrocellulose membranes (Schleicher & Schuell, Germany) using a wet tank transfer system (BioRad). Membranes were blocked 1 h in TBS-T (137 mM NaCl, 2.7 mM KCl, 2.5 mM Tris, pH 7.4, 0.1% Tween-20) containing 5% fetal calf serum, incubated for 2 h at room temperature with primary antibodies (1:1000 dilution), followed by 1 h incubation with secondary antibodies, and revealed with the SuperSignal West Pico (Pierce) chemiluminescence detection system.

## Authors' contributions

SS participated in conceiving and discussing the study, carried out the sequence analysis of ORFs, performed part of the Western blot experiments, oversaw part of the RNA characterization, organized the data, produced the final figures, and wrote most part of the manuscript. FDT participated in conceiving and discussing the study, carried out the genomic DNA sequencing, initiated and carried out part of the characterization of spliced transcripts, provided the sequence data for phylogenetic analyses, produced the VMCH-p8 fusion protein, drafted the figures and helped drafting the manuscript. MJA completed the characterization of the spliced transcripts, performed the analysis of the exon/intron boundaries, and helped preparing the figures and submitting the sequences. ADA carried out the Southern blot experiments and part of the Western blot experiments. RC performed the phylogenetic analyses and appended discussions. JLN conceived and supervised the project, participated in the discussion, wrote part of the manuscript and provided the financial support through grant applications. All authors read and approved the final manuscript.
